# Sober Science

**Published:** 1887-09-10

**Authors:** 


### SOBER SCIENCE.

THE ecclesiastical dovecotes which used to be so alarmingly fluttered about the period of the annual meeting of the British Association, have now enjoyed two or three years of comparative repose. The present and several recent presidents have confined themselves strictly to the business in hand without going out of their way to deal a blow at the Book of Genesis, or to assert the absurdity of miracles old and new. So far as they have manifested any bias at all, it has rather been in favour, not exactly of Catholic or Protestant orthodoxy, but of those broad general principles of morality founded upon religion which are common to all great faiths. For this we, with many other persons who are inclined to "stand in the old ways," at any rate until better ones are discovered, are not ungrateful; though we must admit that what the recent presidential addresses have gained in sobriety they have lost in liveliness, and in power to arouse the popular feelings either of sympathy or of antagonism. Sir Henry Roscoe's presidential address was solid, judicial, and, to the initiated, not without inspiration: but it was certainly caviare to the multitude, and, it must be admitted, dull.

It was not unnatural for the president to take a backward look and to consider all the way the past two or three generations of chemists had been led in the wilderness. For undoubtedly up to the present time much pioneering work has required to be done, and the scientific Joshuas have not always had either an easy or a profitable time of it. They may now be said to have entered upon their Canaan, and to have had time to take a more accurate measurement of the nature and capabilities of their promised land than was possible for them in the earlier days of their partial knowledge in the desert. They know, at any rate to some extent, the nature of their terrestrial abode, and are devising methods of examining the solar and stellar regions which lie beyond. It may be prophesied with some certainty that their knowledge of those further spheres can never be either so extensive or so profound as their knowledge of this on which they for the present reside: unless indeed some of them should carry their passion for chemistry into the condition of immortality, and in that case we know not how many worlds of knowledge may be conquered by their unwearying toil.

But of this world it may be cheerfully admitted they know and have told us a very great deal. Two or three questions occur to the curious and reflecting mind *apropos* of the present state of knowledge in the realm of chemistry. How slow and difficult that knowledge has been of attainment. How partial and incomplete it is now that it is attained. How very small is the difference it has made in the material condition of man! Let no one suppose for a moment that we under-estimate the value of the chemical knowledge which has been won by so much and such noble labours. As mere knowledge, the gain is priceless, whilst in its application to the practical arts, chemistry has done much to render processes precise, to limit waste, to increase resources, and last, but not least, to minister to the necessities of man in times of sickness and deadly peril. All this may be, and is, freely granted; but chemists themselves, and especially those who have attained the highest eminence among them, will admit that they know what disappointment is. With them as with other scientists, knowledge is still relative, not absolute; still of phenomena, not of the thing in itself. Protoplasm is made up of molecules,—true; and molecules can be resolved into atoms,—still true; and atoms under certain conditions will behave themselves in certain ways,—no one doubts it; but what are the atoms, and why do they always under like conditions exhibit like behaviour? Ah, why? Vital force is excluded, even from living things, but when you have spoken of chemical force and chemico-physical processes, are not these terms also to a large extent unknown quantities? They merely imply that we know something about the behaviour of substances that come under observation, but they do not imply—at any rate to those who know—anything of the nature either of finality or of completeness. Where do we stand then in chemistry? Exactly where the biologist stands in biology, and the theologian in theology. Each is on the bosom of a shoreless sea. One with his crucible, his analysis, and his syntheses; another with his scalpel and his microscope; and yet another with his curiosity which is never repressed, his conscience which is never silenced, and his hope which, sometimes crushed beneath mountains of adamant, yet rises again immortal—all of them know and yet do not know; and all of them press forward with inextinguishable ardour to the goal of an absolute knowledge which they shall never reach.

Science teaches now what Christianity taught long ago, that "we see through a glass darkly"; that "we know in part." How apt the phraseology for the purposes of the chemist and the biologist! In these sciences we know what we are capable of knowing, and if we are wise we carry out our knowledge in practice. The man who makes sulphuric acid makes it now by the rules of science. He formerly made it by rule of thumb; but then he did make it. Science simply let in the light of more reason upon that which was already guided by some reason. It is necessary to true progress that the man of science be able to take a just measurement of himself, not only in relation to the affairs with which he is more immediately concerned, but to all other affairs which come within the sphere of human cognisance. Too often he has not done this. He has by toil and infinite pains become master in his own art, and has thereupon jumped to the conclusion that he was master in every other art. As if he should say that because he had been to the summit of the Himalayas therefore he was familiar with everything which could be seen from the top of the Andes. The chemist will admit he is no biologist, and yet he will claim to be a psychologist and a theologian. That, whatever else it may be, is not scientific. On the other hand, the theologian was the first to assert his right to pronounce authoritatively in every region of knowledge, by virtue, not of his labour and research, but of the powers conferred upon him by his church. The war between science and religion is the continuance of an old feud, and the first aggressors were undoubtedly the ecclesiastics. They had a long innings, and made a bad use of it as against scientific men. The latter have had their turn in recent years, and it cannot be said that they have always displayed a strictly scientific temper. On the contrary many of them have been considerably vindictive, and some of them as foolish and unscientific as can well be imagined.

For the present there is a condition of truce. Most of the men of science who have the world's ear are sober and thoughtful persons. They know that they are ignorant of many things as well as that they are learned in some things. The crude speculator is at a discount. The world has found him out and taken his measure. It no longer believes in admirable Crichtons and heaven-born politicians. If a man wants to be taken for an authority he must limit his utterances to those subjects on which he himself has laboured. With the great multitude of men, in theology as in science, there is much to be desired both of increased knowledge and improved temper. But we are glad to think that those who lead the van in these great armies recognise the value of each other's work, and the practical identity of each other's aim. To know well what he does know, and to prove the sincerity of his convictions by the honesty and thoroughness of his work, is the mark of the true man of science, whether his labours lie in the region of matter or of mind.

